# NeoUngulatesDiet: A database of ungulate diets in the Neotropics

**DOI:** 10.1002/ecy.70389

**Published:** 2026-08-18

**Authors:** Laís Lautenschlager, Maria Eduarda Vieira Veloso, Yuri Souza, Letícia Gonçalves Ribeiro, Mateus Melo‐Dias, Sofany Montoya, Rubia Ferreira dos Santos Morini, Bruno Araujo Pinto, Mauro Galetti, Kenneth J. Feeley

**Affiliations:** ^1^ Department of Biology University of Miami Coral Gables Florida USA; ^2^ Centro de Pesquisa em Biodiversidade e Mudanças do Clima (CBioClima), Universidade Estadual Paulista (UNESP), Departamento de Biodiversidade Instituto de Biociências Rio Claro São Paulo Brazil; ^3^ Iniciativa Nacional para a Conservação da Anta Brasileira (INCAB)/Lowland Tapir Conservation Initiative (LCTI) Instituto de Pesquisas Ecológicas (IPÊ) Campo Grande Mato Grosso do Sul Brazil; ^4^ Universidade Estadual de Campinas (UNICAMP), Programa de Pós‐Graduação em Ecologia Instituto de Biologia Campinas São Paulo Brazil; ^5^ Centro Universitário Celso Lisboa Rio de Janeiro Rio de Janeiro Brazil

**Keywords:** data paper, diet, feeding ecology, inventories, Neotropical region, ungulates

## Abstract

Ungulates play a crucial ecological role in tropical forests by influencing various ecosystem processes, including herbivory, seed dispersal and predation, and seedling recruitment. These large mammalian herbivores, including tapirs, camelids, cervids, and peccaries, have a significant impact on plant community structure, composition, and dynamics, especially in the Neotropics. Despite their ecological importance, a systematic understanding of ungulate dietary information has been lacking. The *NeoUngulatesDiet* database addresses this gap by compiling data from a wide range of sources, including peer‐reviewed literature and gray literature such as master's dissertations, Ph.D. theses, and book chapters. *NeoUngulatesDiet* represents a comprehensive compilation of 200 studies conducted between 1976 and 2025. It includes data from 17 countries, 224 plant families, 1135 plant genera, and 2624 plant species consumed by 21 of 28 ungulate species across the Neotropical region. The database comprises a comprehensive repository of consumed plants, including their growth forms, the parts eaten, and associated animal feeding habits. It also provides key information for dietary studies, such as the methodology used, study locations, and the years in which data were collected. This compilation enables researchers to explore the dietary preferences and ecological roles of various ungulate species, which differ in digestive systems, body sizes, and feeding habits. The dataset highlights the need for conservation strategies, particularly for seven understudied Neotropical species for which no dietary information is currently available. Therefore, *NeoUngulatesDiet* serves as a valuable resource for conservation practitioners, supporting the design and implementation of targeted strategies to preserve biodiversity and ecosystem function in the Neotropical region. The data are released under a CC BY 4.0 license. This paper should be cited when the data are used in publications.

## CONFLICT OF INTEREST STATEMENT

The authors declare no conflicts of interest.

## Supporting information


Data S1.

## Data Availability

The dataset is available as Supporting Information in Data S1. Data are also available in Figshare at https://doi.org/10.6084/m9.figshare.29636117.

