# Supplementary material for: NeoUngulatesDiet: A database of ungulate diets in the Neotropics
Source: Ecology. 2026 Aug 18;107(8):e70389. doi: 10.1002/ecy.70389 (PMC13482462; doi:10.1002/ecy.70389)
Supplement: Supplementary file 1 [file ECY-107-e70389-s001.zip › Metadata_S1.pdf]

Data Paper published in *Ecology*, an Ecological Society of America journal

Metadata S1

NeoUngulatesDiet: A database of ungulate diets in the Neotropics

Laís Lautenschlager, Maria Eduarda Vieira Veloso, Yuri Souza, Letícia Gonçalves Ribeiro, Mateus Melo-Dias, Sofany Montoya, Rubia Ferreira dos Santos Morini, Bruno Araujo Pinto, Mauro Galetti, Kenneth J. Feeley

INTRODUCTION

Ungulates, or hoofed mammals, are traditionally classified into two extant orders: Perissodactyla (odd-toed ungulates such as horses, rhinos, and tapirs) and Artiodactyla (even-toed ungulates including deer, pigs, camels, giraffes, cattle, and antelopes). These two orders together encompass 15 extant families, three in Perissodactyla and approximately 12 terrestrial families in Artiodactyla (Mittermeier & Wilson, 2011; Burgin et al., 2018), including an estimated 250 to 280 living species (Mammal Diversity Database, 2024). Terrestrial ungulates are vital contributors to the ecological dynamics of various ecosystems worldwide. This diverse group performs a variety of ecological roles, including the control of plants through trampling and herbivory, nutrient transport, and cycling through their fecal sites, which increase ecosystem heterogeneity due to their social structures and body sizes, and modulate the food web and predator-prey interactions (Mittermeier & Wilson, 2011; Pringle et al., 2023).

Ungulates significantly influence vegetation structure and dynamics through their browsing and grazing habits, often serving as key factors in nutrient movement and cycling and in shaping landscape structure, fire regimes, and forest carbon storage (Found, 2016; Peres et al., 2016; Villar et al., 2021). The interplay between ungulate populations and vegetation availability is crucial, as it can lead to cascading effects on plant communities, soil composition, and overall food web and ecosystem health (Kuijper et al., 2010). Their feeding habits create a heterogeneous environment that promotes biodiversity by allowing different plant species to thrive and supporting habitats for numerous organisms (Costa et al., 2021).

The various threats to ungulate populations, such as hunting and habitat destruction, can lead to detrimental ecological outcomes, including altered species composition and reduced ecosystem resilience (Dahal et al., 2023). Approximately 80% of all wild ungulate species are consumed by humans, used in traditional medicine, and/or used ornamentally (Ripple et al., 2016). Chronic hunting pressures have been shown to severely impact ungulate population densities, significantly disrupting their roles within the ecosystem (Bogoni et al., 2020; Ferreguetti et al., 2023), making them the most locally extirpated group of Neotropical mammals (Bogoni et al., 2020).

Ungulates, such as tapirs, camelids, cervids, and peccaries, play a crucial function in the Neotropics by influencing various ecosystem processes, including herbivory, seed dispersal, and seedling recruitment (Bodmer, 1991). As large mammals, ungulates can also damage plant tissues and physical structures through browsing, trampling, and overturning large amounts of soil (Villar et al., 2021). Consequently, ungulates significantly affect plant community structure, composition, and dynamics (Villar et al., 2020; Souza et al., 2022). For instance, the tapir's browsing habits and seed dispersal can influence the composition and spatial distribution of several plant species within a range of growth forms, which is vital for maintaining biodiversity and forest structure in the Neotropics (Mandujano & Naranjo, 2009; O'Farrill et al., 2013; Souza

et al., 2022). As another example, peccaries, as primary consumers, can alter fruit tree dynamics by preying on seeds (Beck, 2006; Akkawi et al., 2020), thereby affecting plant regeneration (Cullen et al., 2001). Neotropical ungulates exhibit a diverse array of dietary preferences, such as frugivores, browsers, grazers, and mixed-feeders continuum, based on nutritional differences between fruits, browse, and grass (Bodmer, 1990), which reflect their ecological roles and the environments that they inhabit. Core species within this group, such as the white-lipped peccary (*Tayassu pecari*) and various deer species, exhibit highly adaptable feeding habits that are influenced by habitat availability and seasonal changes (Prado, 2013; Jorge et al., 2020). Peccaries are known for their frugivorous diet, which relies heavily on forest cover for optimal foraging (Jorge et al., 2020). In contrast, lowland tapirs are mainly browsers, consuming a variety of foliage, fruits, and seeds of various sizes, making them a significant seed disperser in tropical forest ecosystems (Bodmer, 1991; O'Farrill et al., 2013; Naranjo, 2019). Other Neotropical ungulates, such as llamas (*Lama glama*), are reported to drive soil enrichment through their latrine behavior, and increase the development of aboveground vegetation, as well as transporting viable seeds via endozoochory, likely facilitating dispersal processes in the proglacial plant community (Zimmer et al., 2023).

Studies have highlighted the complexity of dietary habits among Neotropical ungulates, revealing that these animals often exhibit high dietary plasticity, allowing them to exploit available resources across different habitats and consume a wide range of food items. This plasticity demonstrates the ability to adjust their diet in response to seasonal variations and resource availability (Tobler et al., 2009; O'Farrill et al., 2013). For example, in the Cerrado biome, which features heterogeneous habitats of grasslands and scrublands, unique ungulate species have adapted to fire-prone environments, contributing to ecosystem resilience through grazing, seed dispersal, and nutrient cycling (Costa et al., 2021; Valverde et al., 2020). Dietary flexibility plays an important role in the survival of ungulates, allowing them to adapt their feeding strategies to fluctuating environmental pressures, such as habitat modification, especially in fragmented landscapes where resource distribution may be inconsistent (Medici et al., 2022). Moreover, this adaptive capacity extends beyond diet to include flexible habitat use patterns in response to seasonal environmental constraints. For instance, huemul deer (*Hippocamelus bisulcus*) in southern Patagonia shift from high-elevation slopes during the reproductive season to protected forested valleys in autumn and winter following snowfall patterns (Aldridge & Montecinos, 1998; Smith-Flueck, 2000). This pattern illustrates how ungulates integrate multiple selective pressures (e.g., temperature regulation, nutritional quality, and resource availability) to optimize fitness across spatially and temporally heterogeneous landscapes, a capacity that may become increasingly critical as climate change intensifies environmental variability (Figueroa & Corales, 2007).

Consequently, understanding the dietary ecology of Neotropical ungulates not only provides insights into their roles in ecosystems but also informs conservation strategies that aim

to balance their dietary needs with human land-use practices amid ongoing environmental and climate change.

In this context, we introduce the *NeoUngulatesDiet* database – a distinctive data resource that compiles dietary information from a broad range of ungulate species, including many that are often underrepresented in existing datasets or diet-related research, throughout the entire Neotropical region, within the latitude and longitude boundaries of 30° N to 45° S and 35° W to 112° W, respectively (Morrone et al., 2022). In constructing the database, we considered dietary inputs of any recorded plant species consumed by an ungulate involving plant parts (leaves, flowers, fruits, seeds, roots, bark, and pollen (i.e., some studies using palynology to study ungulates' diet)), either in natural or experimental conditions. The *NeoUngulatesDiet* database encompasses the two main ungulate orders (Artiodactyla and Perissodactyla), comprising a total of 21 out of the 28 native and domesticated ungulate species, across four feeding habits (browsers, grazers, frugivores, and mixed feeders) and two digestive systems (polygastric and monogastric). Seven species were not included due to the absence of dietary records in the literature.

Only a few previous databases have focused on plant-animal interactions (from the ATLANTIC series: Bello et al., 2017, frugivory interactions; Iamara-Nogueira et al., 2022, pollination interactions; Boscolo et al., 2023, flower-invertebrate interactions) – most of them with greater attention on particular taxonomic groups and their occurrences (Bovendorp et al., 2017; Lima et al., 2017; Gonçalves et al., 2018; Culot et al., 2019; Souza et al., 2019). In the Neotropics, most published data papers also focus on specific vertebrate taxonomic groups and their spatial distributions. Some of them, from the NEOTROPICAL series: "NEOTROPICAL XENARTHRANS" from Santos et al., 2019; "NEOTROPICAL ALIEN MAMMALS" from Rosa et al., 2020; "NEOTROPICAL CARNIVORES" from Nagy-Reis et al., 2020; "NEOTROPICAL FRESHWATER FISHES" from Tonella et al., 2022; and more recently, "NeoFrugivory" from Fuzessy & Pizo, 2025. The *NeoUngulatesDiet* datapaper differs from the mentioned databases as it covers the dietary aspects of all terrestrial ungulate species in the Neotropical realm through a comprehensive literature search.

It is uncommon to find a database that provides a comprehensive list of the items consumed by animal species or groups, leaving this important information underrepresented. The *NeoUngulatesDiet* addresses this gap by providing the first collated database focused on understanding the diets of ungulates across a broad geographical range. It lists all plant species, growth forms, and plant parts known to be consumed, along with the sampling methodology used, as well as details on location and ecoregion. This resource offers researchers a valuable tool for examining various aspects of the diet and feeding ecology of medium- to large-bodied ungulates. Its wide geographic coverage facilitates the exploration of spatial distribution patterns and region-specific diets across different biogeographic provinces and countries (Figure 1).

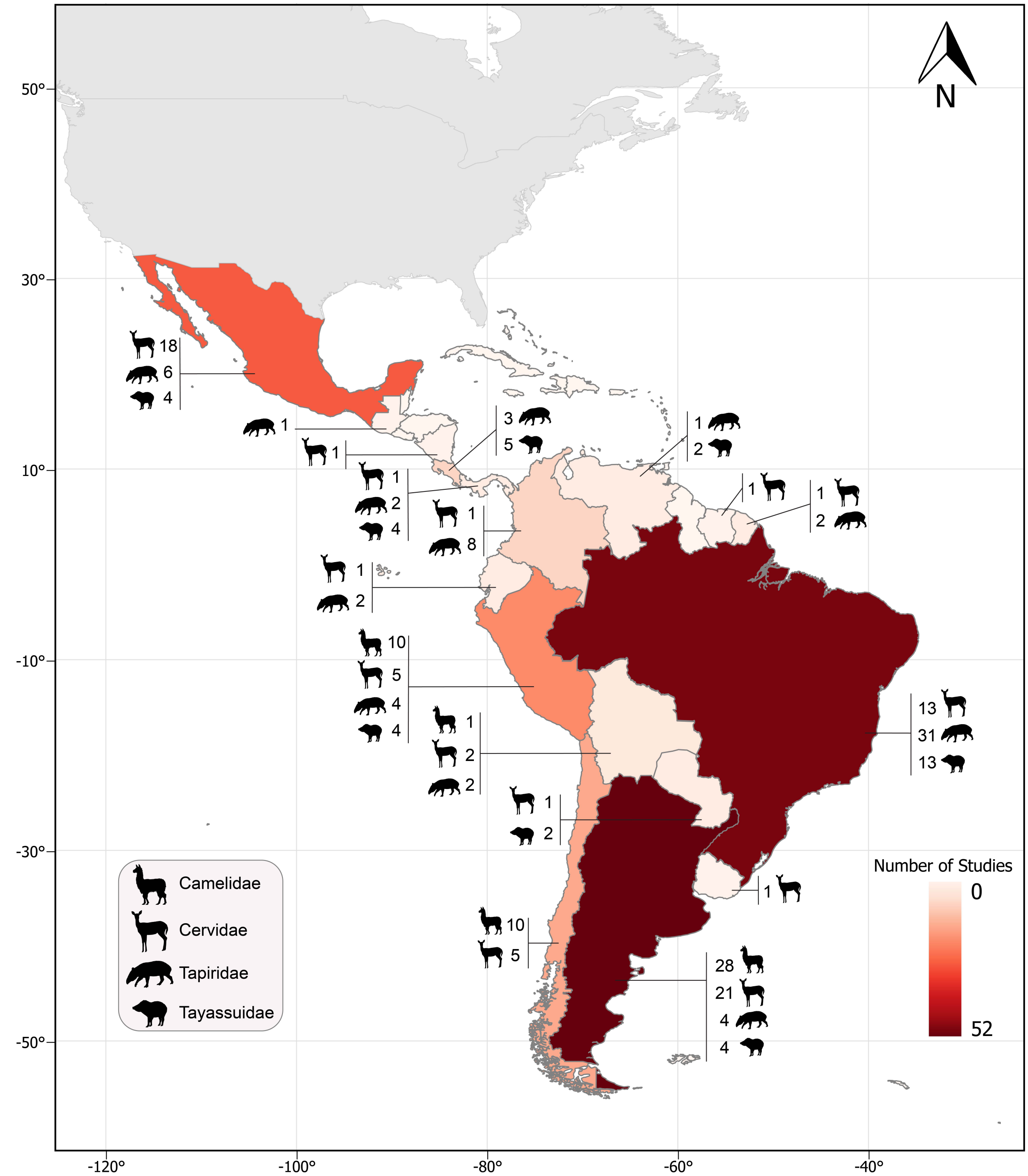

Figure 1. Neotropical region highlighting the diversity of studies and the four main ungulate families (Cervidae, Camelidae, Tapiridae, Tayassuidae). Ungulate families are represented by silhouettes, with study counts displayed alongside. Countries are colored according to the total number of studies (darker colors represent higher counts). Animal silhouettes were sourced from PhyloPic (www.phylopic.org). The following images are available under a CC0 1.0 Universal Public Domain license: *Odocoileus virginianus*, https://www.phylopic.org/images/ccdfd1a8-

f83b-43a2-8307-bf0338290150/odocoileus-virginianus; *Tapirus pinchaque*, https://www.phylopic.org/images/8f6b8802-52f9-4f16-8429-0b86ea4a4aa8/tapirus-pinchaque; *Lama glama*, https://www.phylopic.org/images/aca9eeb8-21bc-4315-a351-17c1c1169801/lama-glama. The image of *Tayassu pecari* is credited to Gabriela Palomo-Munoz (https://www.phylopic.org/images/0ed03276-b356-4092-a4f7-66f4b1842fc4/tayassu-pecari) and available under an Attribution 4.0 International license.

We collected information on 21 out of the 28 native and domesticated native ungulate species from 200 studies conducted between 1976 and 2025 across 17 countries, encompassing 224 unique plant families, 1,135 plant genera, and 2,624 plant species consumed by the studied ungulates. This compilation provides researchers worldwide with valuable data, particularly on severely understudied ungulate species for which no dietary information is available (e.g., *Mazama jucunda*, *M. bricenii*, *M. chunyi*, *M. rufa*, *Passalites nemorivagus*, *Pudella mephistophiles*, and *Pudu puda*, Figure 2).

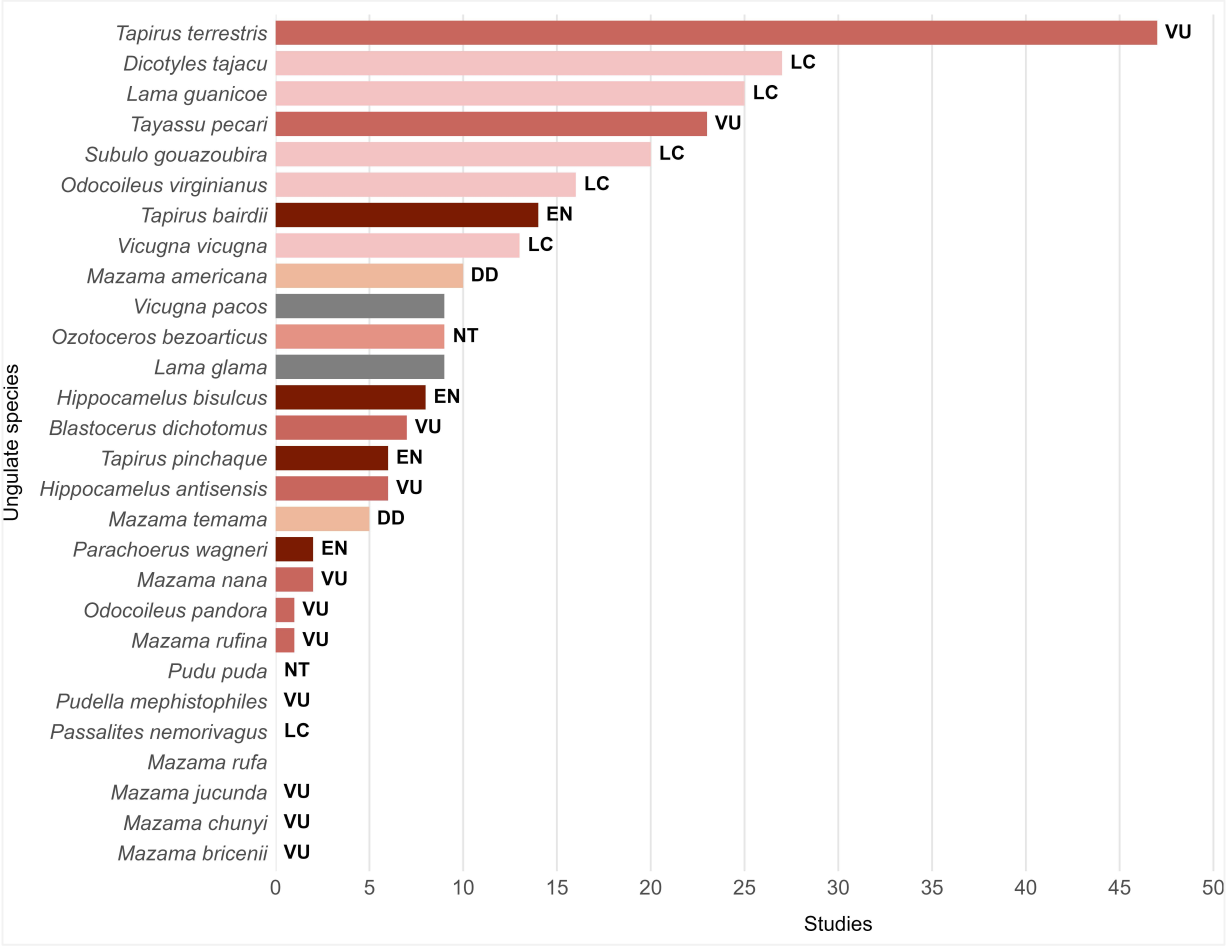

Figure 2. Number of dietary studies and conservation status for each Neotropical ungulate species. Note that no dietary information was found in the literature search for seven species. Four species are Endangered (EN), eleven species are considered Vulnerable (VU), two are Near Threatened (NT), and six are Least Concern (LC). Two species are Data Deficient (DD), and three species do not have a designated conservation status, according to the IUCN Red List of Threatened Species.

Class I. Dataset Descriptors

A. Data set identity:

Title: NeoUngulatesDiet: A database of ungulate diets in the Neotropics

B. Data set identification code: Suggested Dataset Identity Code: NEO_UNGULATES_DIET_DataS1.zip

C. Data set description.

1. **Originators (Principal Investigators):**

Laís Lautenschlager

University of Miami, Department of Biology, Coral Gables, FL, USA, 33146

2. **Abstract:**

Ungulates play a crucial ecological role in tropical forests by influencing various ecosystem processes, including herbivory, seed dispersal and predation, and seedling recruitment. These large mammalian herbivores, including tapirs, camelids, cervids, and peccaries, have a significant impact on plant community structure, composition, and dynamics, especially in the Neotropics. Despite their ecological importance, a systematic understanding of ungulate dietary information has been lacking. The *NeoUngulatesDiet* database addresses this gap by compiling data from a wide range of sources, including peer-reviewed literature and grey literature such as master's dissertations, Ph.D. theses, and book chapters. *NeoUngulatesDiet* represents a comprehensive compilation of 200 studies conducted between 1976 and 2025. It includes data from 17 countries, 224 plant families, 1,135 plant genera, and 2,624 plant species consumed by 21 of 28 ungulate species across the Neotropical region. The database comprises a comprehensive repository of consumed plants, including their growth forms, the parts eaten, and associated animal feeding habits. It also provides key information for dietary studies, such as the methodology used, study locations, and the years in which data were collected. This compilation enables researchers to explore the dietary preferences and ecological roles of various ungulate

species, which differ in digestive systems, body sizes, and feeding habits. The data set highlights the need for conservation strategies, particularly for seven understudied Neotropical species for which no dietary information is currently available. Therefore, *NeoUngulatesDiet* serves as a valuable resource for conservation practitioners, supporting the design and implementation of targeted strategies to preserve biodiversity and ecosystem function in the Neotropical region. The data are released under a CC BY 4.0 license. This paper should be cited when the data are used in publications.

D. Key words/phrases: Ungulates, Diet, Feeding Ecology, Neotropical region, Data paper, Inventories.

(a) The present dataset includes information from 200 studies on the diet of 21 ungulates (out of 28 species) throughout the entire Neotropical Region.

- **Number of ungulate species per family** (after taxonomic update; all 28 species used in the dietary data search):

  Camelidae: *Lama glama*, *Lama guanicoe*, *Vicugna pacos*, *Vicugna vicugna*

  Cervidae: *Blastocerus dichotomus*, *Hippocamelus antisensis*, *Hippocamelus bisulcus*, *Mazama americana*, *Mazama bricenii*, *Mazama chunyi*, *Mazama jucunda*, *Mazama nana*, *Mazama rufa*, *Mazama rufina*, *Mazama temama*, *Odocoileus pandora*, *Odocoileus virginianus*, *Ozotoceros bezoarticus*, *Passalites nemorivagus*, *Pudella mephistophiles*, *Pudu puda*, *Subulo gouazoubira*

  Tapiridae: *Tapirus bairdii*, *Tapirus pinchaque*, *Tapirus terrestris*

  Tayassuidae: *Dicotyles tajacu*, *Parachoerus wagneri*, *Tayassu pecari*

- **Number of ungulates with < 10 diet studies:** 12
  (*Blastocerus dichotomus* (07), *Hippocamelus antisensis* (06), *H. bisulcus* (08), *Lama glama* (09), *Mazama nana* (02), *M. rufina* (01), *M. temama* (05), *Odocoileus pandora* (01), *Ozotoceros bezoarticus* (09), *Parachoerus wagneri* (02), *Tapirus pinchaque* (06), *Vicugna pacos* (09)).

- **Number of ungulates with > 10 diet studies:** 09
  (*Dicotyles tajacu* (27), *Lama guanicoe* (25), *Mazama americana* (10), *Odocoileus virginianus* (16), *Subulo gouazoubira* (20), *Tapirus bairdii* (14), *T. terrestris* (47), *Tayassu pecari* (23), *Vicugna vicugna* (13)).

- **Number of ungulates without any diet studies:** 07
  (*Mazama jucunda*, *M. bricenii*, *M. chunyi*, *M. rufa*, *Passalites nemorivagus*, *Pudella mephistophiles*, *Pudu puda*).

- **Year (start - final):** 1976 - 2025

  [1976, 1978, 1981, 1982, 1983, 1984, 1985, 1988, 1989, 1990, 1991, 1992, 1993, 1994, 1995, 1996, 1997, 1998, 1999, 2000, 2001, 2002, 2003, 2004, 2005, 2006, 2007, 2008, 2009, 2010, 2011, 2012, 2013, 2014, 2015, 2016, 2017, 2018, 2019, 2020, 2021, 2022, 2023, 2024, 2025].

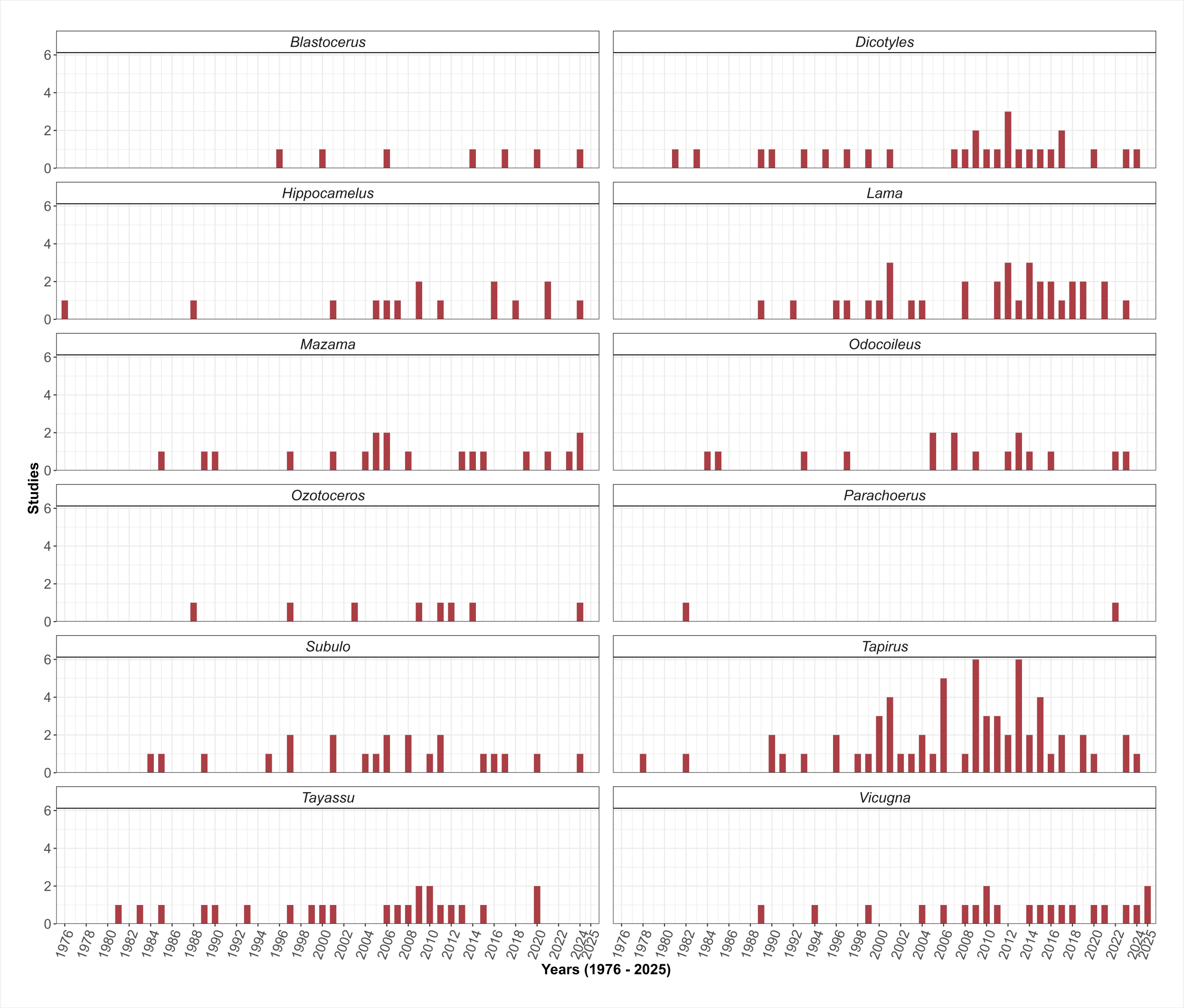

Figure 3. Yearly number of studies per genus of Neotropical ungulates from 1976 to 2025.

Ungulate feeding behaviors in the Neotropics vary significantly across geographic and climatic gradients, reflecting the region's ecological diversity. In tropical rainforests such as the Amazon and Atlantic Forest, where rainfall is abundant, and plant productivity remains high

year-round, many ungulates display frugivorous and browsing habits, relying on fruits, leaves, and understory vegetation (Bodmer, 1990). In contrast, in seasonally dry ecosystems such as the Cerrado, Caatinga, and Puna (high-Andean Mountain grasslands), ungulates often adopt grazing or mixed-feeding strategies, adapting to the seasonal availability of grasses and drought-resistant plants (Janis, 2008). Wetter highland environments, such as the Páramos, characterized by cooler temperatures and specialized plant communities, typically support ungulates that predominantly graze on herbaceous vegetation (Muñoz et al., 2013; Ramos et al., 2021). These behavioral adaptations are closely tied to variations in resource availability driven by elevation, temperature, rainfall, and seasonality—highlighting the ecological plasticity of Neotropical ungulates in response to their environment. Even within the same biome, dietary strategies can vary considerably among species, depending on factors such as body size, digestive physiology, and foraging behavior. These differences underscore the combined influence of climatic and temporal conditions (e.g., rainfall, temperature, seasonality) and species-specific traits on feeding ecology throughout the Neotropics.

- **Number of plant species consumed** (after taxonomic update)**:** 2,624.
- **Number of plant genera consumed** (after taxonomic update)**:** 1,135.
- **Number of plant families consumed** (after taxonomic update)**:** 224.

The distribution of dispersal events across plant families reveals preferences for certain plant families within different ungulate families, highlighting the varying ecological roles played by different ungulates in shaping plant community dynamics. For instance, Poaceae and Asteraceae were largely consumed by Camelidae and Cervidae (grazers and browsers/mixed feeders). In contrast, Arecaceae and Fabaceae were mostly consumed by Tapiridae and Tayassuidae species (browsers and frugivores). Cervidae consumed the widest range of distinct plant species, followed by Tapiridae, Camelidae, and Tayassuidae, respectively (Figure 4).

- **Number of studies per country (for 21 ungulate species):** Argentina (52), Bolivia (04), Brazil (48), Chile (15), Colombia (08), Costa Rica (08), Ecuador (03), French Guiana (03), Guatemala (01), Mexico (27), Nicaragua (01), Panama (03), Paraguay (03), Peru (20), Suriname (01), Uruguay (01), Venezuela (03).

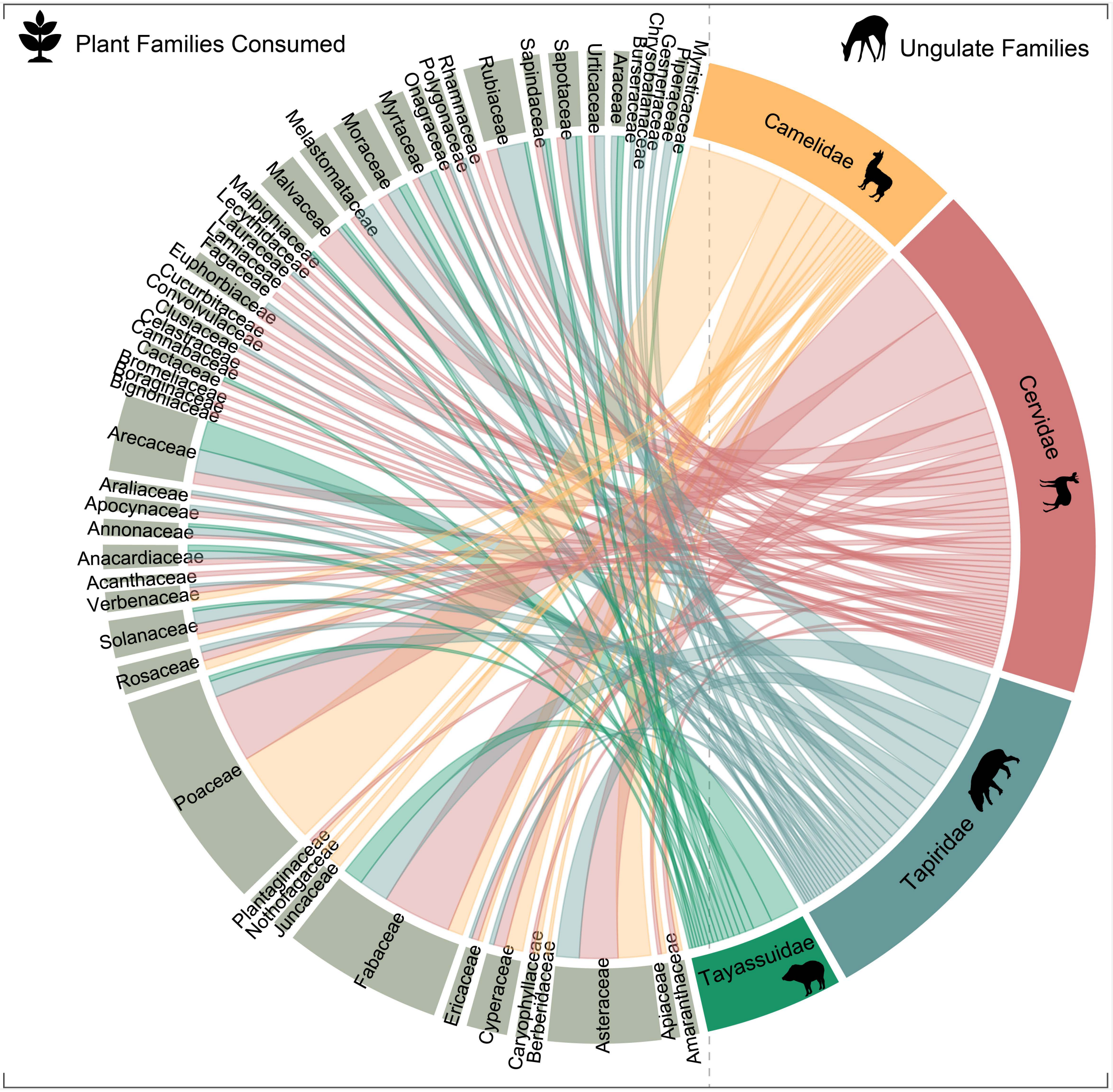

Figure 4. Chord diagram representing the most common plant families consumed by each ungulate family: Camelidae, Cervidae, Tapiridae, and Tayassuidae. Only plant families with over 10 consumption records were included to facilitate clearer visualization. Seedling silhouette created by Laís Lautenschlager. Animal silhouettes were sourced from PhyloPic (www.phylopic.org). The following images are available under a CC0 1.0 Universal Public Domain license: *Odocoileus virginianus*, https://www.phylopic.org/images/ccdfd1a8-f83b-43a2-8307-bf0338290150/odocoileus-virginianus; *Tapirus pinchaque*, https://www.phylopic.org/images/8f6b8802-52f9-4f16-8429-0b86ea4a4aa8/tapirus-pinchaque; *Lama glama*, https://www.phylopic.org/images/aca9eeb8-21bc-4315-a351-17c1c1169801/lama-glama. The image of *Tayassu pecari* is credited to Gabriela Palomo-Munoz

(https://www.phylopic.org/images/0ed03276-b356-4092-a4f7-66f4b1842fc4/tayassu-pecari) and available under an Attribution 4.0 International license.

(b) The dataset also includes information on plant parts consumed by the ungulates, as well as the growth forms according to GBIF and POWO. Only vegetative parts consumed by ungulate species were reported in the dataset (bark, flower, fruit, root, seed, leaf, or a combination thereof). Numbers in parentheses represent the counts for each record in the dataset within all ungulate species and studies:

- Plant parts consumed: Bark (03), Flower (40), Fruit (1,114), Fruit_flower (94), Fruit_root (01), Fruit_seed (99), Leaf (3,309), Leaf_bark (01), Leaf_flower (17), Leaf_fruit (67), Leaf_root (02), Root (27), Seed (185), Seed_leaf (05), and Seed_root (02).
- Plant growth forms: Climber (235), Herb (orchid, cacti, cushion, fern, lichen, bromeliad) (1,519), Grass (635), Palm (237), Shrub (1,089), and Tree (1,443).

(c) Several methods were used by the authors to obtain ungulate dietary information. Microhistological analysis was the most used method among researchers (38.7%), followed by focal observation (22.9%) and fecal analysis (15.7%, Figure 5). Numbers in parentheses represent the number of distinct references according to the method applied. Some studies combined different methods in the same sampling design:

- Camera_trap (01), DNA Metabarcoding (02), Fecal_analysis (28), Fecal_and_focal_observation (04), Fecal_and_microhistological_analysis (01), Fecal_and_stomach_analysis (03), Focal_observation (35), Interview (03), Isotope_analysis (01), Microhistological_analysis (66), Palynology (04), Stomach_analysis (08), Stomach_analysis_and_focal_observation (01), Stomach_and_microhistological_analysis (01).

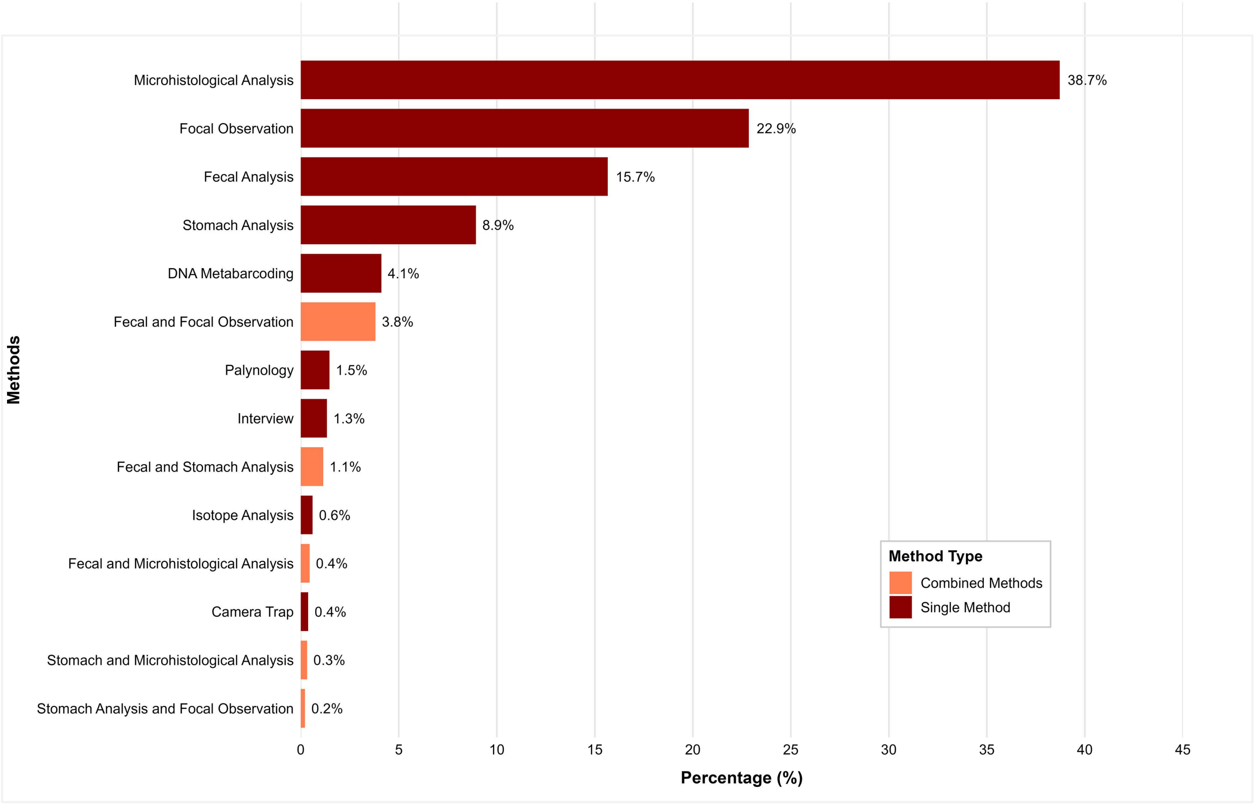

Figure 5. Methods used by researchers to study the diets of Neotropical ungulate species. Red bars show single-method studies; orange bars indicate studies combining two methods.

Class II. Research origin descriptors

A. Overall project description:

1. Identity: *NeoUngulatesDiet: A Database of Ungulate Diets in the Neotropics*

This compilation includes all available information from 200 studies reporting on the diets of 21 ungulates across 28 species in the entire Neotropical region. Data was obtained from peer-reviewed papers, master's dissertations, Ph.D. theses, and book chapters. We avoided using information from literature review papers.

2. Originators:

The *NeoUngulatesDiet project* is an initiative designed to provide substantial, freely accessible data to ecologists, biologists, and other researchers in the fields of animal feeding behavior and plant-animal sciences. The database provides valuable information to scientists investigating ecological interactions, dietary preferences, and feeding ecology across a wide range of terrestrial herbivorous and frugivorous ungulates across the entire Neotropical region.

The data compilation was led by Laís Lautenschlager, with contributions from Maria Eduarda Vieira Veloso, Yuri Souza, Letícia Gonçalves, Mateus Melo-Dias, Sofany Montoya, Rubia Ferreira dos Santos Morini, and Bruno Araujo Pinto. Laís Lautenschlager, Mauro Galetti, and Kenneth Feeley conceptualized the project. All co-authors reviewed the Metadata.

Laís Lautenschlager[1], Maria Eduarda Vieira Veloso[2], Yuri Souza[3], Letícia Gonçalves Ribeiro[2], Mateus Melo-Dias[2], Sofany Montoya[1], Rubia Ferreira dos Santos Morini[4], Bruno Araujo Pinto[5], Mauro Galetti[2], and Kenneth Feeley[1].

[1] University of Miami, Department of Biology, Coral Gables, FL, USA

[2] CBioClima, Universidade Estadual Paulista (UNESP), Departamento de Biodiversidade, Instituto de Biologia, Rio Claro, SP, Brazil

[3] Iniciativa Nacional para a Conservação da Anta Brasileira (INCAB)/Lowland Tapir Conservation Initiative (LCTI), Instituto de Pesquisas Ecológicas (IPÊ), Campo Grande, MS, Brazil

[4] Universidade Estadual de Campinas (UNICAMP), Programa de Pós-Graduação em Ecologia, Instituto de Biologia, Campinas, SP, Brazil

[5] Centro Universitário Celso Lisboa, Rio de Janeiro, RJ, Brazil

3. **Period of study**: 1976 - May 2025
   Compiled studies published and publicly available until May 2025.

4. **Objectives:**

Our primary objective in compiling this dataset was to summarize all available information on the diets of 21 ungulate species (out of a total of 28 species) across the Neotropical region. Our dataset represents the first comprehensive attempt to understand what is known about the dietary preferences and feeding ecology of ungulates across a wide geographic range, encompassing diverse bioregions, climatic variations, and feeding habits. In this dataset, we considered data from peer-reviewed papers, master's dissertations, Ph.D. theses, and book chapters.

5. **Abstract**:

Ungulates play a crucial ecological role in tropical forests by influencing various ecosystem processes, including herbivory, seed dispersal and predation, and seedling recruitment. These large mammalian herbivores, including tapirs, camelids, cervids, and peccaries, have a significant impact on plant community structure, composition, and dynamics, especially in the Neotropics. Despite their ecological importance, a systematic understanding of ungulate dietary

information has been lacking. The *NeoUngulatesDiet* database addresses this gap by compiling data from a wide range of sources, including peer-reviewed literature and grey literature such as master's dissertations, Ph.D. theses, and book chapters. *NeoUngulatesDiet* represents a comprehensive compilation of 200 studies conducted between 1976 and 2025. It includes data from 17 countries, 224 plant families, 1,135 plant genera, and 2,624 plant species consumed by 21 of 28 ungulate species across the Neotropical region. The database comprises a comprehensive repository of consumed plants, including their growth forms, the parts eaten, and associated animal feeding habits. It also provides key information for dietary studies, such as the methodology used, study locations, and the years in which data were collected. This compilation enables researchers to explore the dietary preferences and ecological roles of various ungulate species, which differ in digestive systems, body sizes, and feeding habits. The data set highlights the need for conservation strategies, particularly for seven understudied Neotropical species for which no dietary information is currently available. Therefore, *NeoUngulatesDiet* serves as a valuable resource for conservation practitioners, supporting the design and implementation of targeted strategies to preserve biodiversity and ecosystem function in the Neotropical region. The data are released under a CC BY 4.0 license. This paper should be cited when the data are used in publications.

6. **Sources of funding**:

The compilation of this dataset was supported by several grants, fellowships, and scholarships: LL and MG received the research grant "Universal Project," process number 405839/2021–9, from the Brazilian National Council for Scientific and Technological Development (CNPq) during 2021-2024. RFSM received a research grant from the São Paulo Research Foundation (FAPESP), process number 2023/02722-5, from 2023 to 2024.

B. Specific subproject description

1. Site description

Data were collected from various sites across the Neotropical region, which spans a wide range of elevations (0–5,000 meters above sea level), and encompasses the tropical and subtropical regions of Central and South America (30° N to 45° S, and 35° W to 112° W). The Neotropics, one of the world's eight biogeographic realms (Olson et al., 2001), is a complex mosaic of large urban centers, extensive agricultural lands, and forested areas, and exhibits a wide range of climatic conditions, with mean annual rainfall ranging from 1,000 to 10,000 mm and significant variation between the wettest and driest months. Mean annual temperatures vary from 10°C in highland areas to 28°C in lowland tropical forests, showing substantial variation between the warmest and coldest periods.

The dataset spans a diverse array of geographic regions and biomes, including tropical rainforests (Amazon, Atlantic Forest, and Mesoamerican rainforests), tropical and subtropical

dry forests (Cerrado savanna, Caatinga, and Central American Dry Forests), tropical and subtropical grasslands and shrublands (Llanos and Rupestrian Fields), montane grasslands and shrublands (Páramos and the Andes), and xeric shrublands (Gran Chaco). The Neotropics also retain a significant portion of global biodiversity, considered the most species-rich regions on Earth (Antonelli et al., 2018).

2. Experimental or sampling design

We compiled studies reporting geographic locations (i.e., coordinates) and methodological approaches, along with a list of plant species consumed by 21 of 28 Neotropical ungulate species, as listed in the *Handbook of the Mammals of the World – Hoofed Mammals* (Mittermeier & Wilson, 2011). Non-native (introduced) ungulate species were excluded to ensure that the dataset reflects the dietary patterns of native species within their natural habitats and ecological contexts. Dietary information from introduced or highly domesticated ungulates is often strongly influenced by human management, altered habitats, supplementary feeding, and novel plant assemblages, which can obscure natural feeding patterns and limit comparability within native species (e.g., *Sus scrofa, Equus spp*., *Bos spp*., *Camelus spp*., *Bubalus bubalis*, *Ovis aries*, *Capra hircus*, *Axis axis*, *Dama dama*, *Cervus elaphus*, and *Odocoileus hemionus*).

After selecting the species, we first conducted a systematic literature search in the Web of Science (WoS) database (*www.webofknowledge.com*), using all available databases and collections. Searches were performed using species-specific strings in three languages:

English: “[scientific species name]” AND “diet”, OR “habit”, OR “feeding”, OR “feeding ecology”, OR “nutrition”, OR “foraging ecology”, OR “foraging behavior”;

Spanish: “[*scientific species name*]” AND “dieta” OR “hábito” OR “alimentación” OR “ecología de la alimentación (ecología alimentaria)” OR “nutrición” OR “ecología del forrajeo” OR “comportamiento de forrajeo”;

Portuguese: “[*scientific species name*]” AND “dieta” OR “hábito alimentar” OR “alimentação” OR “ecologia alimentar” OR “nutrição” OR “ecologia de forrageamento” OR “comportamento de forrageamento”;

We selected the useful filters offered by the WoS platform (e.g., the different databases (all), publication years (all), document types (all), research areas (all), publication/source titles (all), countries/regions (all), languages (mainly English, Spanish, Portuguese), and others).

Following the Web of Science search and initial screening, we conducted complementary Google Scholar searches to retrieve additional records not indexed in WoS. Google Scholar searches used species-specific strings combining the scientific name with diet-related keywords (e.g., “diet,” “feeding ecology,” “foraging behavior,” “food habits,” “nutrition,” and “foraging

ecology”). Equivalent searches were performed in Portuguese and Spanish to enhance coverage of regional literature. Scientific names were placed within quotation marks to ensure exact matches. Results were sorted by relevance, and up to the first 200 records returned per species – keyword search were screened based on title and abstract, with full texts assessed when necessary. Duplicate records across databases were identified by comparing titles, authorship, publication year, and DOI (when available) and were removed prior to eligibility assessment. Studies were included when they reported primary dietary information for free-ranging Neotropical ungulates and excluded if they lacked diet data, focused solely on captive populations, occurred outside the Neotropics, addressed non-native species, or consisted exclusively of general reviews without original data.

Across both databases, we compiled peer-reviewed articles, regional journal publications, conference proceedings, and grey literature (e.g., theses and dissertations). When necessary, we included both former and most updated scientific species names (e.g., *Mazama pandora* and *Odocoileus pandora* (Gutiérrez et al., 2017); *Catagonus wagneri* and *Parachoerus wagneri* (Parisi-Dutra et al., 2017); *Pecari tajacu* and *Dicotyles tajacu* (Acosta et al., 2020); *Mazama bororo* and *Mazama jucunda* (Mantellatto et al., 2021); *Mazama gouazoubira* and *Subulo gouazoubira* (Bernegossi et al., 2023); *Mazama nemorivaga* and *Passalites nemorivagus* (Morales-Donoso et al., 2023); *Pudu mephistophiles* and *Pudella mephistophiles* (Barrio, 2025). Taxonomic updates followed the most recent literature available at the time of the search (last updated on June 16, 2025) (Figure 6).

Because database compilation spanned multiple search phases, early screening records were not fully archived. Identification and screening counts were therefore reconstructed from available search outputs and compilation tables and should be interpreted as best-faith estimates. Google Scholar searches relied on relevance-ranked results, and screening depth was standardized to the first records returned per search, which may introduce selection bias. Despite multilingual searches and grey literature inclusion, some sources may remain undetected.

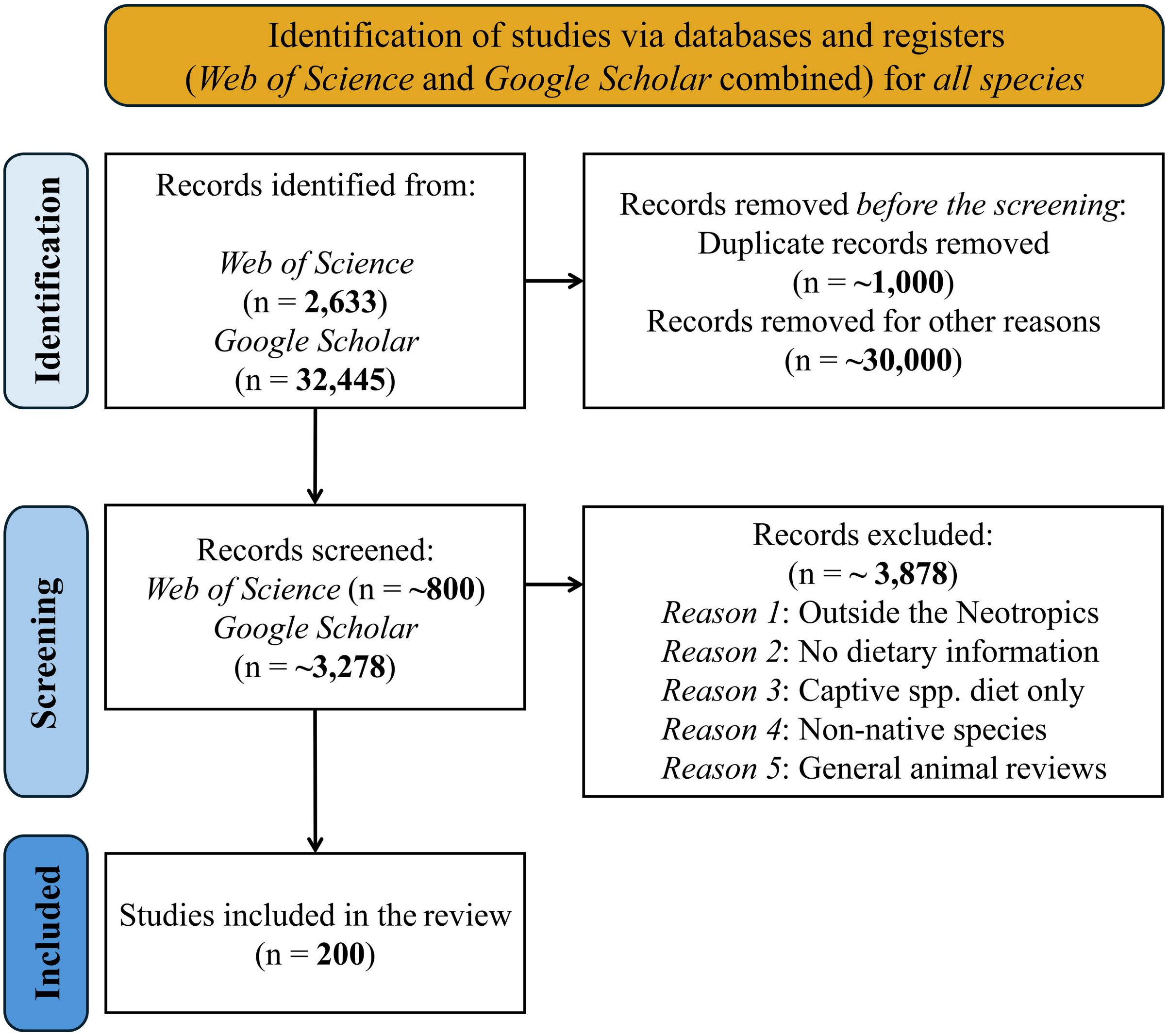

Figure 6. PRISMA-adapted flow diagram summarizing the literature search and selection process for studies included in the *NeoUngulatesDiet* dataset. Records were identified through Web of Science and Google Scholar databases using species-specific search strings. Because the database compilation spanned multiple search phases, exact counts from the earliest screening stages were not consistently archived. Therefore, initial screening totals and removals were reconstructed from available records. Reconstructed values should be interpreted as best-faith estimates of the search workflow rather than exact historical counts.

3. Research methods

After compiling the studies, we skimmed the abstracts, methods, and results of each paper to identify potential information relevant to our data paper. When the study did not address the consumed plant at the genus or species taxonomic level, it was automatically eliminated. For

the studies retained after this initial screening, we searched for information in figures, tables, results, and/or supplementary material. If the data associated with a paper were not available either as supplementary material or in an online repository, it was also eliminated. We also excluded studies conducted outside the Neotropical region (e.g., some articles on white-tailed deer (*Odocoileus virginianus*) or collared peccaries (*Dicotyles tajacu*), whose distributions extend into North America).

Based on the methodologies described in the selected manuscripts, we accepted nine main methods: focal observation, fecal analysis, stomach/rumen analysis, microhistological analysis, interviews, isotope analysis, DNA metabarcoding, camera trapping, and palynology (pollen analysis), or combinations thereof. We excluded literature reviews and general animal reviews, opting instead to use original research articles that provided details on study location and sampling design. Feeding experiments were also excluded unless the animals were fed with native or local plant species, indicated as “Free-ranging” or “Captive” studies.

We updated the taxonomic information on plant species' scientific names from the original source, using the *taxize* package and the “tropicos” database, through R software (R Core Team, 2025) (last verified on July 15, 2025). This package interacts with a suite of web application programming interfaces (API) for taxonomic tasks. Some of the services supported include NCBI E-utilities, Encyclopedia of Life, Global Biodiversity Information Facility (GBIF), and others (Chamberlain et al., 2020). In a few cases where taxonomic names were not available, we manually searched for their updated names in the Plants of the World Online (POWO) and GBIF repositories. At least four authors cross-checked the database to ensure consistency in the information entered, including location names, geographic coordinates, species names, and the methodology classification, as previously described.

4. **Project personnel:**

Laís Lautenschlager, Maria Eduarda Vieira Veloso, Yuri Souza, Letícia Gonçalves Ribeiro, Mateus Melo-Dias, Sofany Montoya, Rubia Ferreira dos Santos Morini, Bruno Araujo Pinto, Mauro Galetti, and Kenneth Feeley.

Class III. Data set status and accessibility

A. Status

1. **Latest update**: July 21, 2025
2. **Latest archive date**: July 24, 2025
3. **Metadata status**: July 21, 2025

4. **Data verification**: The data were manually checked, and inconsistent values (e.g., typos, incorrect spelled scientific names) were either corrected or removed from the database.

B. Accessibility

1. **Storage location and medium**: In addition to this data paper published in Ecology, data files are also stored in the Figshare repository. Please refer to the "*NEO_UNGULATES_DIET_DataS1*" folder (doi: 10.6084/m9.figshare.29636117).

2. **Contact persons**: Laís Lautenschlager. University of Miami, Department of Biology, Coral Gables, FL, USA. The Jungle Biology Lab (room #190). (lais.lrodrigues@miami.edu; rodrigues.eco12@gmail.com)

3. **Copyright restrictions**: None.

4. **Proprietary restrictions**: There are no limitations on data usage, and no permission is required. However, we kindly ask users to cite this paper when using the data in publications and to inform us about the data's application: Laís Lautenschlager (lais.lrodrigues@miami.edu; rodrigues.eco12@gmail.com)

5. **Costs**: None.

Class IV. Data structural descriptors

A. Data set file

1. **Identity:** Data_S1.zip (compressed files and folders).

2. **Size:** Included in the "Data_S1" folder (115 KB):

   NeoUngulatesDiet_References.csv, 44 KB

   NeoUngulatesDiet_Dataset.csv, 1,913 KB

   NeoUngulatesDiet_Animal_info.csv, 5 KB

   Format and storage mode: All comma-separated files (.csv) are included in a compressed folder (.zip).

3. **Header information**: See column descriptors in section B (Variable information) – Tables 1, 2 and 3.

4. **Alphanumeric attributes**: Mixed.

5. **Special characters/fields:** None.

6. **Authentication procedures**: None.

B. Variable information

C. Data anomalies: Missing information was classified as “NA”.

Class V. Supplemental descriptors

A. Data acquisition

1. **Data forms or acquisition methods**: The acquisition method is fully described in section Class II, B, 1-3.

2. **Location of completed data forms:** None.

3. **Data entry verification procedures**: The core team systematically checked data entry to ensure that no errors were carried over to the final version of the dataset. We also standardized column names, content, and coordinate format. In addition, we cleaned any special character (e.g., ç, ñ, á, â, ã, é, í, ó, ú, ü).

B. Quality assurance/quality control procedures: **Related materials**: Individual data sets were manually reviewed and aggregated into a single dataset. Manual checks were performed on missing, duplicate, and erroneous entries.

C. Related materials: All data is available in “Data_S1.zip” (115 KB) file.

D. Computer programs and data-processing algorithms: The data were entered using Microsoft Excel, cleaned, analyzed, and analyzed using R software (R Core Team, 2025). The maps were created using ArcGIS Pro (3.5.2) (ESRI, 2024).

E. Archiving

1. **Archival procedures:** In addition to this data paper published in Ecology, data files are also stored in the Figshare repository. Please refer to the “*NEO_UNGULATES_DIET_DataS1*” folder (doi: 10.6084/m9.figshare.29636117).

2. **Redundant archival sites:** None.

F. Publications and results: None.

G. History of data set usage

1. **Data request history**: None.
2. **Data set update history**: None.
3. **Review history:** None.
4. **Questions and comments from secondary users:** None.

Acknowledgments

This paper is part of the NEOTROPICAL, ATLANTIC, BRAZIL, and AMAZONIA series of data papers in *Ecology*. LL acknowledges the Brazilian National Council for Scientific and Technological Development (CNPq) for the research grant “Universal Project” (#405839/2021–9) and additional funding from the Department of Biology, University of Miami. LL also acknowledges all authors for their contributions to conceptual ideas, data compilation, and tabulation.

Literature Citations

Acosta, L.E., Garbino, G.S.T., Gasparini, G.M. & Dutra, R.P. (2020). Unraveling the nomenclatural puzzle of the collared and white-lipped peccaries (Mammalia, Cetartiodactyla, Tayassuidae). *Zootaxa*, 4851 (1), 60–80. https://doi.org/10.11646/zootaxa.4851.1.2

Akkawi, P., Villar, N., Mendes, C. P., & Galetti, M. (2020). Dominance hierarchy on palm resource partitioning among Neotropical frugivorous mammals. *Journal of Mammalogy*, 101(3), 697-709. https://doi.org/10.1093/jmammal/gyaa052

Aldridge, D. & Montecinos, L. (1998). Avances en la conservación del Huemul (*Hippocamelus bisulcus*) en Chile. In: V. Valverde (Ed.) La conservación de la fauna nativa de Chile, Logros y perspectivas. Ministerio de Agricultura, CONAF, Chile 178: 133-149 pp.

Antonelli, A., Zizka, A., Carvalho, F. A., Scharn, R., Bacon, C. D., Silvestro, D., & Condamine, F. L. (2018). Amazonia is the primary source of Neotropical biodiversity. *Proceedings of the National Academy of Sciences*, 115(23), 6034-6039. https://doi.org/10.1073/pnas.17138191

Barrio, J. (2025). *Pudella mephistophiles* (Artiodactyla: Cervidae). *Mammalian Species*, 57(1042), seaf001. https://doi.org/10.1093/mspecies/seaf001

Beck, H. (2006). A review of peccary-palm interactions and their ecological ramifications across the Neotropics. *Journal of Mammalogy*, *87*(3), 519-530. https://doi.org/10.1644/05-MAMM-A-174R1.1

Bello, C., Galetti, M., Montan, D., Pizo, M. A., Mariguela, T. C., Culot, L., ... & Jordano, P. (2017). Atlantic frugivory: a plant–frugivore interaction data set for the Atlantic Forest. *Ecology*, 98(6), 1729. https://doi.org/10.1002/ecy.1818

Bernegossi, A. M., Borges, C. H. D. S., Sandoval, E. D. P., Cartes, J. L., Cernohorska, H., Kubickova, S., ... & Duarte, J. M. B. (2023). Resurrection of the genus Subulo for the gray brocket deer, with designation of a neotype. *Journal of Mammalogy*, 104(3), 619-633. https://doi.org/10.1093/jmammal/gyac068

Bodmer, R. E. (1990). Responses of ungulates to seasonal inundations in the Amazon floodplain. *Journal of Tropical Ecology*, 6(2), 191-201. https://doi.org/10.1017/S0266467400004314

Bodmer, R. E. (1990). Ungulate frugivores and the browser-grazer continuum. *Oikos*, 319-325. https://doi.org/10.2307/3565960

Bodmer, R. E. (1991). Strategies of seed dispersal and seed predation in Amazonian ungulates. *Biotropica*, 255-261. https://doi.org/10.2307/2388202

Bodmer, R. E., & Ward, D. (2006). Frugivory in large mammalian herbivores. *CONSERVATION BIOLOGY SERIES-CAMBRIDGE-*, *11*, 232. https://doi.org/10.1017/CBO9780511617461.010

Bogoni, J. A., Peres, C. A., & Ferraz, K. M. (2020). Extent, intensity, and drivers of mammal defaunation: a continental-scale analysis across the Neotropics. *Scientific reports*, 10(1), 14750. https://doi.org/10.1038/s41598-020-72010-w

Boscolo, D., Nobrega Rodrigues, B., Ferreira, P. A., Lopes, L. E., Tonetti, V. R., Reis dos Santos, I. C., ... & Glebya Maciel Quirino, Z. (2023). Atlantic flower–invertebrate interactions: A data set of occurrence and frequency of floral visits. *Ecology*, 104(3), e3900. https://doi.org/10.1002/ecy.3900

Bovendorp, R. S., Villar, N., de Abreu-Junior, E. F., Bello, C., Regolin, A. L., Percequillo, A. R., & Galetti, M. (2017). Atlantic small-mammal: a dataset of communities of rodents and marsupials of the Atlantic forests of South America. *Ecology*, 98(8), 2226. https://doi.org/10.1002/ecy.1893

Burgin, C. J., Colella, J. P., Kahn, P. L., & Upham, N. S. (2018). How many species of mammals are there? *Journal of Mammalogy*, 99(1), 1–14. https://doi.org/10.1093/jmammal/gyx147

Chamberlain, S., Szoecs, E., Foster, Z., Arendsee, Z., Boettiger, C., Ram, K., ... & Grenié, M. (2020). *taxize*: Taxonomic information from around the web. *R package version 0.9, 92*. https://github.com/ropensci/taxize.

Costa, H., Benchimol, M., & Peres, C. (2021). Wild ungulate responses to anthropogenic land use: a comparative pantropical analysis. *Mammal Review*, 51(4), 528-539. https://doi.org/10.1111/mam.12252

Cullen, L., Bodmer, E., & Valladares-Pádua, C. (2001). Ecological consequences of hunting in Atlantic forest patches, São Paulo, Brazil. *Oryx*, 35(02), 137. https://doi.org/10.1046/j.1365-3008.2001.00163.x

Culot, L., Pereira, L. A., Agostini, I., de Almeida, M. A. B., Alves, R. S. C., Aximoff, I., ... & Galetti, M. (2019). ATLANTIC-PRIMATES: A dataset of communities and occurrences of primates in the Atlantic Forests of South America. *Ecology*, 100(1), e02525. https://doi.org/10.1002/ecy.2525

Dahal, U., Raut, N., Dhital, A., Mainali, S., Gautam, D., & Tripathi, S. (2023). Impacts of livestock grazing on wild ungulate habitat in the khata corridor, bardiya, nepal. International *Journal of Forestry Research*, 2023, 1-11. https://doi.org/10.1155/2023/3981111

Danell, K., Bergström, R., Duncan, P., & Pastor, J. (Eds.). (2006). Large herbivore ecology, ecosystem dynamics and conservation (Vol. 11). Cambridge University Press. ISBN: 9780521536875.

ESRI (2024). *ArcGIS Pro (Version 3.5.2)* [Computer software]. Environmental Systems Research Institute. https://www.esri.com/en-us/arcgis/products/arcgis-pro/overview

Figueroa, R., & Corales, E. S. (2007). Hábitat y dieta del huemul (Hippocamelus bisulcus) en el corredor biológico Nevados de Chillán-Laguna Laja: la importancia de la flora andina para la conservación de un cérvido en peligro de extinción. *Revista Chagual*, *5*, 21-32

Found, R. (2016). Managing large herbivores in protected areas. *Global Journal of Ecology*, 1(1), 001-011. https://doi.org/10.17352/gje.000001

Ferreguetti, A., Allemand, M. M., Pereira-Ribeiro, J., Tomas, W. M., Rocha, C. F., Bergallo, H. G., & Miller, D. A. (2023). Don't shoot! How poaching may affect richness, biomass, and occupancy of non-volant mammals in an Atlantic Forest protected area, Brazil. *Research Square*. https://doi.org/10.21203/rs.3.rs-2681323/v1

Fuzessy, L., & Pizo, M. A. (2025). NeoFrugivory: A comprehensive database for frugivory interactions and functional traits in the Neotropics. *Ecology*, *106*(3), e70049. https://doi.org/10.1002/ecy.70049

GBIF Secretariat: GBIF Backbone Taxonomy. https://doi.org/10.15468/39omei. [16 June 2025].

Gonçalves, F., Bovendorp, R. S., Beca, G., Bello, C., Costa-Pereira, R., Muylaert, R. L., ... & Galetti, M. (2018). ATLANTIC MAMMAL TRAITS: a data set of morphological traits of mammals in the Atlantic Forest of South America. 99 (2), 498. https://doi.org/10.1002/ecy.2106

Gutiérrez, E. E., Helgen, K. M., McDonough, M. M., Bauer, F., Hawkins, M. T., Escobedo-Morales, L. A., ... & Maldonado, J. E. (2017). A gene-tree test of the traditional taxonomy of American deer: the importance of voucher specimens, geographic data, and dense sampling. *ZooKeys,* (697), 87. https://doi.org/10.3897/zookeys.697.15124

Hofmann, R. R. (1989). Evolutionary steps of ecophysiological adaptation and diversification of ruminants: a comparative view of their digestive system. *Oecologia*, 78(4), 443-457. https://doi.org/10.1007/BF00378733

Iamara-Nogueira, J., Targhetta, N., Allain, G., Gambarini, A., Pinto, A. R., Rui, A. M., ... & Buzato, S. (2022). ATLANTIC POLLINATION: a data set of flowers and interaction with nectar-feeding vertebrates from the Atlantic Forest. *Ecology*, 103(2), e03595. https://doi.org/10.1002/ecy.3595

ITIS (2025). Retrieved [June, 16, 2025], from the Integrated Taxonomic Information System (ITIS), www.itis.gov, CC0. https://doi.org/10.5066/F7KH0KBK

Kuijper, D., Cromsigt, J., Jędrzejewska, B., Miścicki, S., Churski, M., Jędrzejewski, W., … & Kweczlich, I. (2010). Bottom-up versus top-down control of tree regeneration in the białowieża primeval forest, Poland. *Journal of Ecology*, 98(4), 888-899. https://doi.org/10.1111/j.1365-2745.2010.01656.x

Janis, C. (2008). An evolutionary history of browsing and grazing ungulates. In *The ecology of browsing and grazing* (pp. 21-45). Berlin, Heidelberg: Springer Berlin Heidelberg. https://doi.org/10.1007/978-3-540-72422-3_2

Jorge, M., Bradham, J., Keuroghlian, A., Oshima, J., & Ribeiro, M. (2020). Permeability of neotropical agricultural lands to a key native ungulate—are well-connected forests important?. *Biotropica*, 53(1), 201-212. https://doi.org/10.1111/btp.12861

Lima, F., Beca, G., Muylaert, R. L., Jenkins, C. N., Perilli, M. L., Paschoal, A. M. O., ... & Galetti, M. (2017). ATLANTIC-CAMTRAPS: A dataset of medium and large terrestrial mammal communities in the Atlantic Forest of South America. *Ecology*, 98(11), 2979. https://doi.org/10.1002/ecy.1998

Mammal Diversity Database. (2024). *Mammal Diversity Database*. American Society of Mammalogists. https://www.mammaldiversity.org/

Mantellatto, A. M. B., González, S., & Duarte, J. M. B. (2021). Cytochrome b sequence of the *Mazama americana jucunda* Thomas, 1913 holotype reveals *Mazama bororo* Duarte, 1996 as its junior synonym. *Genetics and Molecular Biology,* 45(1), e20210093. https://doi.org/10.1590/1678-4685-GMB-2021-0093

Mandujano, S., & Naranjo, E. (2009). Ungulate biomass across a rainfall gradient: a comparison of data from neotropical and palaeotropical forests and local analyses in Mexico. *Journal of Tropical Ecology*, 26(1), 13-23. https://doi.org/10.1017/s0266467409990411

Mittermeier, R. A., & Wilson, D. E. (2011). Handbook of the mammals of the world: Vol. 2: hoofed mammals. Lynx Edicions. ISBN: 978-84-96553-77-4

Morales-Donoso, J. A., Vacari, G. Q., Bernegossi, A. M., Sandoval, E. D. P., Peres, P. H. F., Galindo, D. J., ... & Duarte, J. M. B. (2023). Revalidation of Passalites Gloger, 1841 for the Amazon brown brocket deer *P. nemorivagus* (Cuvier, 1817) (Mammalia, Artiodactyla, Cervidae). *ZooKeys*, 1167, 241. https://doi.org/10.3897/zookeys.1167.100577

Morrone, J. J., Escalante, T., Rodríguez-Tapia, G., Carmona, A., Arana, M., & Mercado-Gómez, J. D. (2022). Biogeographic regionalization of the Neotropical region: New map and shapefile. *Anais da Academia Brasileira de Ciências*, 94, e20211167. https://doi.org/10.1590/0001-3765202220211167

Muñoz, A. E., & Simonetti, J. A. (2013). Diet of guanaco in sheep-free rangeland in Tierra del Fuego, Chile. *Ciencia e Investigación Agraria*, 40(1), 185–191. http://dx.doi.org/10.4067/S0718-16202013000100016

Nagy-Reis, M., Oshima, J. E. D. F., Kanda, C. Z., Palmeira, F. B. L., de Melo, F. R., Morato, R. G., ... & Lopes, C. M. (2020). NEOTROPICAL CARNIVORES: a data set on carnivore distribution in the Neotropics. *Ecology*, 101(11), e03128. https://doi.org/10.1002/ecy.3128

O'Farrill, G., Galetti, M., & Campos-Arceiz, A. (2013). Frugivory and seed dispersal by tapirs: an insight on their ecological role. *Integrative zoology*, *8*(1), 4-17. https://doi.org/10.1111/j.1749-4877.2012.00316.x

Olson, D. M., Dinerstein, E., Wikramanayake, E. D., Burgess, N. D., Powell, G. V., Underwood, E. C., ... & Kassem, K. R. (2001). Terrestrial Ecoregions of the World: A New Map of Life on Earth: A new global map of terrestrial ecoregions provides an innovative tool for conserving biodiversity. *BioScience*, *51*(11), 933-938. https://doi.org/10.1641/0006-3568(2001)051[0933:TEOTWA]2.0.CO;2

Parisi-Dutra, R., Casali, D. D. M., Missagia, R. V., Gasparini, G. M., Perini, F. A., & Cozzuol, M. A. (2017). Phylogenetic systematics of peccaries (Tayassuidae: Artiodactyla) and a

classification of South American tayassuids. *Journal of Mammalian Evolution*, 24, 345-358. https://doi.org/10.1007/s10914-016-9347-8

Peres, C. A., Emilio, T., Schietti, J., Desmoulière, S. J., & Levi, T. (2016). Dispersal limitation induces long-term biomass collapse in overhunted Amazonian forests. *Proceedings of the National Academy of Sciences*, *113*(4), 892-897. https://doi.org/10.1073/pnas.1516525113

Prado, H. (2013). Feeding ecology of five neotropical ungulates: a critical review. *Oecologia Australis*, 17(4), 459-473. https://doi.org/10.4257/oeco.2013.1704.02

Pringle, R. M., Abraham, J. O., Anderson, T. M., Coverdale, T. C., Davies, A. B., Dutton, C. L., ... & Veldhuis, M. P. (2023). Impacts of large herbivores on terrestrial ecosystems. *Current Biology*, *33*(11), R584-R610. https://doi.org/10.1016/j.cub.2023.04.024

Ramos, R., Chirinos, D., Contreras, J., & Curasma, J. (2021). Evaluación de especies de pastizales consumidos por alpacas madres y tuis (Vicugna pacos) en época seca en bofedales de Yauyos. *Journal of High Andean Research*, 23(2), 103–110. http://dx.doi.org/10.18271/ria.2021.219

R Core Team. (2025). R: A language and environment for statistical computing (Version 4.4.0). R Foundation for Statistical Computing. https://www.R-project.org/

Ripple, W. J., Abernethy, K., Betts, M. G., Chapron, G., Dirzo, R., Galetti, M., ... & Young, H. (2016). Bushmeat hunting and extinction risk to the world's mammals. *Royal Society open science*, *3*(10), 160498. https://doi.org/10.1098/rsos.160498

Rosa, C. A. D., Ribeiro, B. R., Bejarano, V., Puertas, F. H., Bocchiglieri, A., Barbosa, A. L. D. S., ... & Carvalho, D. R. (2020). Neotropical Alien Mammals: a data set of occurrence and abundance of alien mammals in the Neotropics. *Ecology*, 101(11), e03115. https://doi.org/10.1002/ecy.3115

Santos, P. M., Bocchiglieri, A., Chiarello, A. G., Paglia, A. P., Moreira, A., de Souza, A. C., ... & Fischer, E. (2019). NEOTROPICAL XENARTHRANS: a data set of occurrence of xenarthran species in the Neotropics. *Ecology*, 100(7), e02663. https://doi.org/10.1002/ecy.2663

Smith-Flueck, J. A. (2000). La situación actual del huemul patagónico. In: Díaz N y J Smith-Flueck (eds). El Huemul Patagónico: un misterioso cérvido al borde de la extinción. Editorial LOLA., Buenos Aires, Argentina 170: 67-150 pp.

Souza, Y., Gonçalves, F., Lautenschlager, L., Akkawi, P., Mendes, C., Carvalho, M. M., ... & Galetti, M. (2019). ATLANTIC MAMMALS: a data set of assemblages of medium-and large-sized mammals of the Atlantic Forest of South America. *Ecology*, *100*(10), e02785. https://doi.org/10.1002/ecy.2785

Souza, Y., Villar, N., Zipparro, V., Nazareth, S., & Galetti, M. (2022). Large mammalian herbivores modulate plant growth form diversity in a tropical rainforest. *Journal of Ecology*, *110*(4), 845-859. https://doi.org/10.1111/1365-2745.13846

Tonella, L. H., Ruaro, R., Daga, V. S., Garcia, D. A. Z., Vitorino, O. B., Lobato-de Magalhães, T., ... & Carmassi, G. R. (2023). NEOTROPICAL FRESHWATER FISHES: A dataset of occurrence and abundance of freshwater fishes in the Neotropics. *Ecology*, 104(4), e3713. https://doi.org/10.1002/ecy.3713

Villar, N., Siqueira, T., Zipparro, V., Farah, F., Schmaedecke, G., Hortenci, L., ... & Galetti, M. (2020). The cryptic regulation of diversity by functionally complementary large tropical forest herbivores. *Journal of Ecology*, 108(1), 279-290. https://doi.org/10.1111/1365-2745.13257

Villar, N., Paz, C., Zipparro, V., Nazareth, S., Bulascoschi, L., Bakker, E. S., & Galetti, M. (2021). Frugivory underpins the nitrogen cycle. *Functional Ecology*, 35(2), 357-368. https://doi.org/10.1111/1365-2435.13707

Wilson, D. E., & Mittermeier, R. A. (2011). Handbook of the mammals of the world, volume 2: hoofed mammals (pp. 886-886). Barcelona: Lynx Ediciones. ISBN: 8496553779Zimmer, A., Beach, T., Riva Regalado, S., Salcedo Aliaga, J., Cruz Encarnación, R., & Anthelme, F. (2023). Llamas (*Llama glama*) enhance proglacial ecosystem development in Cordillera Blanca, Peru. *Scientific Reports*, 13(1), 15936. https://doi.org/10.1038/s41598-023-41458-x

Tables

Table 1. Variable identity names and descriptions for the file "NeoUngulatesDiet_References.csv".

Variable/Column	Description	Type of data (values)
Code	Unique identification code for each paper/data source. This column provides the 'key' connecting the sources listed here to the data in Table 2 (NeoUngulatesDiet_Dataset.csv).	Text and numeric (REF1 - REF200)
References	Source reference following APA format. This column provides the complete reference connecting the sources from the "Reference code", "Citation", and "DOI_links" columns in Table 2 (NeoUngulatesDiet_Dataset.csv).<br>APA format:<br>Author's last name, initials, publication year in parentheses, the title of the article in sentence case, the journal name in italics and title case, volume number in italics, issue number in parentheses (if applicable), page range.	Text<br><br>(Bogoni, J. A., Peres, C. A., & Ferraz, K. M. (2020). Extent, intensity, and drivers of mammal defaunation: a continental-scale analysis across the Neotropics. *Scientific Reports*, 10(1), 14750.)

Table 2. Variable names and descriptions for the file "NeoUngulatesDiet_Dataset.csv".

Variable/Column	Description	Type of data (values)
ID	Numerical ID for each row of the spreadsheet (continuous)	Numeric<br>(#1 - #5320)
Reference_code	Numerical code unique identification for each Reference used in "Citation" and "DOI_links" columns (refer to the file: NeoUngulatesDiet_References.csv)	Text and numeric<br>(REF1 - REF200)
Animal_Order	Animal taxonomic Order (Artiodactyla or Perissodactyla)	Text<br>(e.g., Perissodactyla)
Animal_Family	Animal taxonomic Family (Cervidae, Camelidae, Tayassuidae, or Tapiridae)	Text<br>(e.g., Tayassuidae)
Animal_Genus	Animal taxonomic Genus (13 possible animal genera)	Text<br>(e.g., Tayassu)
Animal_Species	Animal taxonomic Species scientific name (28 possible animal species), separated by an underscore symbol	Text<br>(e.g., Tayassu_pecari)
Site	Study site description or location name, according to the source (in English, Spanish, or Portuguese)	Text<br>(e.g., Ibera_National_Park)
State/Provincia/ Department	Study site description of the State, Provincia, or Department, according to the original source (in English, Spanish, or Portuguese)	Text<br>(e.g., Corrientes)
Country	Study site description of the country, according to the original source	Text<br>(e.g., Argentina)
Elevation_masl	Study site description of the Elevation (m.a.s.l units), according to the original source. According to the range, it could include a hyphen (-) between the elevation values.	Numeric<br>(e.g., 50-250)
Biome_paper	Study site description of the biome or vegetation, according to the original source (in English, Spanish, or Portuguese), separated by an underscore symbol	Text<br>(e.g., Deciduous_forest)
Latitude	Study site Latitude (Geographic coordinate system, World Geodetic System 1984 (WGS 84)), in Decimal degrees	Numeric<br>(e.g., -22.37892196)

Longitude	Study site Longitude (Geographic coordinate system, World Geodetic System 1984 (WGS 84)), in Decimal degrees	Numeric<br>(e.g., -49.67974274)
Study_ Environment	Environment in which the animal individuals from the original Reference were studied (Free-Ranging or Captive).	Text<br>(e.g., Free-ranging)
Method	Method used to obtain the data from the original Reference, following the original description from the source, but standardized by the main author of this data paper in English, separated by an underscore symbol.<br>(camera_trap, DNA_metabarcoding, fecal_analysis, fecal_and_focal_observation, fecal_and_microhistological_analysis, fecal_and_stomach_analysis, focal_observation, interview, isotope_analysis, microhistological_analysis, palynology (pollen analysis), stomach_analysis, stomach_analysis_and_focal_observation, stomach_and_microhistological_analysis)	Text<br>(e.g., microhistological_ analysis)
Data_collection_ Year	The year in which the data was collected (not when it was published), according to the original source. When > 2 years, separated by a hyphen (-)	Numeric<br>(e.g., 2013 - 2015).
Body_Mass_kg	Animal body mass, according to the book "Handbook of the Mammals of the World: Hoofed Mammals" (Mittermeier & Wilson, 2011). According to the range, it could include a hyphen (-) between the weight values	Numeric, unit: Kilograms<br>(e.g., 30-130)
Digestive_tract	Animal digestive tract (Monogastric or Polygastric), according to Wilson & Mittermeier (2011).	Text<br>(e.g., Polygastric)
Diet	Animal dietary habits, standardized by the main author of this data paper according to 04 categories (in English) (browser, frugivore, grazer, mixed_feeder), separated by an underscore symbol. Dietary categories according to Bodmer (1990), Bodmer & Ward (2006), Hofmann (1989), Danell et al. (2006), and Wilson & Mittermeier (2011).	Text<br>(e.g., Browser)
Plant_Family	Consumed plant Family, according to the original source	Text<br>(e.g., Fabaceae)

Plant_Genus	Consumed plant Genus, according to the original source	Text<br>(e.g., Solanum)
Plant_Species	Consumed plant Species scientific name, according to the original source	Text<br>(e.g., *Spondias purpurea*)
Part_Consumed	Parts of the plant consumed by the ungulate species, according to the original source, standardized by the main author of this data paper, according to 15 categories (in English) (bark, flower, fruit, fruit_flower, fruit_root, fruit_seed, leaf, leaf_bark, leaf_flower, leaf_fruit, leaf_root, root, seed, seed_leaf, seed_root), separated by an underscore symbol	Text<br>(e.g, seed)
Growth_form	Consumed Plant growth form, according to the original Reference, and standardized by the main author of this data paper according to 06 categories (in English) (palm, tree (> 3m height), shrub (< 3 m height), herb (orchid, cacti, cushion, fern, lichen, bromeliad), climber (vines, lianas), grass	Text<br>(e.g., herb)
Corrected_Spp_ Family	Consumed plant Family, after taxonomic update according to the '*taxize*' package in R (Chamberlain et al., 2020), Plants of the World Online and GBIF databases. Last verification on July 15, 2025.	Text<br>(e.g., Arecaceae)
Corrected_Spp_ Genus	Consumed plant Genus, after taxonomic update according to the '*taxize*' package in R ("tropicos" database) (Chamberlain et al., 2020), Plants of the World Online, and GBIF databases. Last verification on July 15, 2025.	Text<br>(e.g., Euterpe)
Corrected_Spp_ Names	Consumed plant Species names, after taxonomic update according to the '*taxize*' package in R (Chamberlain et al., 2020), Plants of the World Online, and GBIF databases. Last verification on July 15, 2025.	Text<br>(e.g., *Euterpe edulis*)
Citation	Citation for the Reference (02 authors, last names separated by "AND" (e.g., "SILVA_AND_SILVEIRA_2025"); 03 or more authors, first author last name + "ETAL" (e.g., "RODRIGUES_ETAL_2025"))	Text<br>(e.g., Rodrigues_etal_2002)
DOI_links	DOI links or weblinks are available to access the original Reference/source.	Text<br>(e.g., 10.19136/era.a11n2.3981)

Table 3. Variable names and descriptions for the file “NeoUngulatesDiet_Animal_info.csv”.

Variable/Column	Description	Type of data (values)
Animal_Order	Animal taxonomic Order (Artiodactyla or Perissodactyla)	Text (e.g., Perissodactyla)
Animal_Family	Animal taxonomic family (Cervidae, Camelidae, Tayassuidae, or Tapiridae)	Text (e.g., Tayassuidae)
Animal_Species	Animal taxonomic species scientific name (28 possible animal species), separated by an underscore symbol	Text (e.g.,Tayassu_pecari)
Updated_Animal_ Species	Animal taxonomic species scientific name updated according to Integrated Taxonomic Information System (ITIS, 2025) and GBIF Backbone Taxonomy (GBIF, 2025), and recent scientific publications.	Text (e.g., Dicotyles_tajacu)
Common_name_ English	Animal common name, in English	Text (e.g., Collared peccary)
Common_name_ Portuguese	Animal common name, in Portuguese	Text (e.g., Cateto)
Common_name_ Spanish	Animal common name, in Spanish	Text (e.g., Ciervo de los Pantanos)
Body_Mass_kg	Animal body mass, according to the book “Handbook of the Mammals of the World: Hoofed Mammals” (Mittermeier & Wilson, 2011). According to the range, it could include a hyphen (-) between the weight values	Numeric, unit: Kilograms (e.g., 30-130)
Digestive_tract	Animal digestive tract (Monogastric or Polygastric), according to Wilson & Mittermeier (2011).	Text (e.g., Polygastric)

Diet	Animal dietary habits, standardized by the main author of this data paper according to 04 categories (in English) (browser, frugivore, grazer, mixed_feeder), separated by an underscore symbol. Dietary categories according to Bodmer (1990), Bodmer & Ward (2006), Hofmann (1989), Danell et al. (2006), and Wilson & Mittermeier (2011).	Text<br>(e.g., browser_frugivore)
IUCN_status	Animal threat status according to the IUCN Red List of Threatened Species (https://www.iucnredlist.org/)	Text<br>(e.g., Vulnerable)
IUCN_acronym	Animal threat status acronym, according to the IUCN Red List of Threatened Species (https://www.iucnredlist.org/)	Text<br>(e.g., VU)
